# Supplementary material for: Modeling glioblastoma heterogeneity as a dynamic network of cell states
Source: Mol Syst Biol. 2021 Sep 16;17(9):e10105. doi: 10.15252/msb.202010105 (PMC8444284; doi:10.15252/msb.202010105)
Supplement: Supplementary file 5 — Source Data for Figure 3 [file MSB-17-e10105-s001.zip › Figure3A_sourcedata/GSEA_3065/hallmarks_state5.GseaPreranked.1623416302988/HALLMARK_APOPTOSIS.html]

Details for gene set HALLMARK\_APOPTOSIS[GSEA]

|  || Dataset | state5 |
| Phenotype | NoPhenotypeAvailable |
| Upregulated in class | na\_neg |
| GeneSet | HALLMARK\_APOPTOSIS |
| Enrichment Score (ES) | -0.29346487 |
| Normalized Enrichment Score (NES) | -1.0167861 |
| Nominal p-value | 0.43142146 |
| FDR q-value | 0.72637534 |
| FWER p-Value | 1.0 |
Table: GSEA Results Summary

  

Fig 1: Enrichment plot: HALLMARK\_APOPTOSIS      
 Profile of the Running ES Score & Positions of GeneSet Members on the Rank Ordered List

  

| PROBE | GENE SYMBOL | GENE\_TITLE | RANK IN GENE LIST | RANK METRIC SCORE | RUNNING ES | CORE ENRICHMENT || 1 | PLAT |  |  | 37 | 0.714 | 0.0278 | No |
| 2 | APP |  |  | 38 | 0.712 | 0.0596 | No |
| 3 | TIMP1 |  |  | 109 | 0.518 | 0.0749 | No |
| 4 | CLU |  |  | 162 | 0.474 | 0.0902 | No |
| 5 | DNAJC3 |  |  | 192 | 0.444 | 0.1068 | No |
| 6 | BCAP31 |  |  | 235 | 0.415 | 0.1206 | No |
| 7 | EMP1 |  |  | 258 | 0.404 | 0.1363 | No |
| 8 | CD44 |  |  | 378 | 0.349 | 0.1385 | No |
| 9 | ERBB3 |  |  | 411 | 0.337 | 0.1499 | No |
| 10 | TIMP2 |  |  | 451 | 0.325 | 0.1601 | No |
| 11 | BRCA1 |  |  | 522 | 0.305 | 0.1658 | No |
| 12 | F2R |  |  | 568 | 0.293 | 0.1739 | No |
| 13 | TNFRSF12A |  |  | 615 | 0.282 | 0.1813 | No |
| 14 | RHOT2 |  |  | 660 | 0.273 | 0.1885 | No |
| 15 | IGF2R |  |  | 705 | 0.260 | 0.1952 | No |
| 16 | PPT1 |  |  | 744 | 0.251 | 0.2022 | No |
| 17 | WEE1 |  |  | 919 | 0.222 | 0.1925 | No |
| 18 | IFNGR1 |  |  | 983 | 0.213 | 0.1949 | No |
| 19 | SLC20A1 |  |  | 1035 | 0.206 | 0.1984 | No |
| 20 | EBP |  |  | 1041 | 0.206 | 0.2070 | No |
| 21 | TAP1 |  |  | 1077 | 0.202 | 0.2121 | No |
| 22 | SC5D |  |  | 1094 | 0.199 | 0.2192 | No |
| 23 | TIMP3 |  |  | 1191 | 0.186 | 0.2167 | No |
| 24 | PSEN1 |  |  | 1217 | 0.183 | 0.2221 | No |
| 25 | ERBB2 |  |  | 1309 | 0.170 | 0.2194 | No |
| 26 | IFITM3 |  |  | 1332 | 0.168 | 0.2244 | No |
| 27 | MMP2 |  |  | 1824 | 0.125 | 0.1747 | No |
| 28 | CFLAR |  |  | 1828 | 0.125 | 0.1800 | No |
| 29 | CASP2 |  |  | 2006 | 0.111 | 0.1650 | No |
| 30 | MCL1 |  |  | 2055 | 0.108 | 0.1644 | No |
| 31 | XIAP |  |  | 2072 | 0.107 | 0.1674 | No |
| 32 | TGFB2 |  |  | 2112 | 0.104 | 0.1677 | No |
| 33 | BTG2 |  |  | 2174 | 0.100 | 0.1653 | No |
| 34 | PSEN2 |  |  | 2293 | 0.092 | 0.1561 | No |
| 35 | DNM1L |  |  | 2401 | 0.085 | 0.1479 | No |
| 36 | SPTAN1 |  |  | 2534 | 0.077 | 0.1365 | No |
| 37 | IRF1 |  |  | 2926 | 0.056 | 0.0949 | No |
| 38 | PTK2 |  |  | 2987 | 0.052 | 0.0905 | No |
| 39 | ANKH |  |  | 3098 | 0.047 | 0.0802 | No |
| 40 | CTNNB1 |  |  | 3417 | 0.031 | 0.0458 | No |
| 41 | MADD |  |  | 4170 | -0.000 | -0.0389 | No |
| 42 | ATF3 |  |  | 4225 | -0.002 | -0.0449 | No |
| 43 | PPP2R5B |  |  | 4284 | -0.005 | -0.0512 | No |
| 44 | PAK1 |  |  | 4352 | -0.008 | -0.0584 | No |
| 45 | DPYD |  |  | 4382 | -0.010 | -0.0612 | No |
| 46 | PLPPR4 |  |  | 4411 | -0.011 | -0.0639 | No |
| 47 | PPP3R1 |  |  | 4413 | -0.011 | -0.0636 | No |
| 48 | DFFA |  |  | 4481 | -0.013 | -0.0705 | No |
| 49 | CDK2 |  |  | 4489 | -0.013 | -0.0707 | No |
| 50 | SATB1 |  |  | 4505 | -0.014 | -0.0718 | No |
| 51 | CCND2 |  |  | 4539 | -0.016 | -0.0748 | No |
| 52 | BMP2 |  |  | 4770 | -0.025 | -0.0996 | No |
| 53 | ADD1 |  |  | 5036 | -0.034 | -0.1279 | No |
| 54 | RNASEL |  |  | 5101 | -0.037 | -0.1335 | No |
| 55 | RARA |  |  | 5104 | -0.037 | -0.1320 | No |
| 56 | GADD45B |  |  | 5106 | -0.037 | -0.1305 | No |
| 57 | LEF1 |  |  | 5255 | -0.042 | -0.1453 | No |
| 58 | DDIT3 |  |  | 5335 | -0.046 | -0.1521 | No |
| 59 | CASP4 |  |  | 5377 | -0.048 | -0.1546 | No |
| 60 | BCL10 |  |  | 5406 | -0.049 | -0.1556 | No |
| 61 | NEDD9 |  |  | 5611 | -0.058 | -0.1760 | No |
| 62 | TOP2A |  |  | 5722 | -0.063 | -0.1856 | No |
| 63 | ISG20 |  |  | 5757 | -0.064 | -0.1865 | No |
| 64 | GSR |  |  | 5778 | -0.065 | -0.1859 | No |
| 65 | CREBBP |  |  | 5871 | -0.068 | -0.1932 | No |
| 66 | ENO2 |  |  | 5895 | -0.069 | -0.1927 | No |
| 67 | CYLD |  |  | 5903 | -0.070 | -0.1904 | No |
| 68 | CASP7 |  |  | 5942 | -0.072 | -0.1914 | No |
| 69 | CDKN1B |  |  | 5996 | -0.074 | -0.1941 | No |
| 70 | FDXR |  |  | 6009 | -0.074 | -0.1921 | No |
| 71 | PDCD4 |  |  | 6228 | -0.086 | -0.2128 | No |
| 72 | ROCK1 |  |  | 6236 | -0.087 | -0.2097 | No |
| 73 | RHOB |  |  | 6287 | -0.090 | -0.2114 | No |
| 74 | DAP3 |  |  | 6378 | -0.094 | -0.2173 | No |
| 75 | HMOX1 |  |  | 6561 | -0.106 | -0.2331 | No |
| 76 | PMAIP1 |  |  | 6872 | -0.128 | -0.2623 | No |
| 77 | SOD2 |  |  | 6961 | -0.134 | -0.2662 | No |
| 78 | TXNIP |  |  | 7037 | -0.140 | -0.2684 | No |
| 79 | CASP3 |  |  | 7082 | -0.144 | -0.2669 | No |
| 80 | CDC25B |  |  | 7102 | -0.146 | -0.2626 | No |
| 81 | CASP6 |  |  | 7206 | -0.156 | -0.2672 | No |
| 82 | RELA |  |  | 7298 | -0.167 | -0.2700 | No |
| 83 | BCL2L1 |  |  | 7308 | -0.168 | -0.2635 | No |
| 84 | CAV1 |  |  | 7575 | -0.200 | -0.2845 | Yes |
| 85 | GSN |  |  | 7578 | -0.201 | -0.2757 | Yes |
| 86 | BTG3 |  |  | 7602 | -0.204 | -0.2692 | Yes |
| 87 | GADD45A |  |  | 7633 | -0.208 | -0.2633 | Yes |
| 88 | ETF1 |  |  | 7748 | -0.225 | -0.2661 | Yes |
| 89 | JUN |  |  | 7930 | -0.262 | -0.2747 | Yes |
| 90 | HMGB2 |  |  | 7973 | -0.271 | -0.2674 | Yes |
| 91 | SQSTM1 |  |  | 8044 | -0.284 | -0.2625 | Yes |
| 92 | SAT1 |  |  | 8162 | -0.314 | -0.2617 | Yes |
| 93 | BID |  |  | 8165 | -0.315 | -0.2478 | Yes |
| 94 | DNAJA1 |  |  | 8208 | -0.328 | -0.2379 | Yes |
| 95 | BNIP3L |  |  | 8266 | -0.344 | -0.2289 | Yes |
| 96 | DAP |  |  | 8313 | -0.357 | -0.2181 | Yes |
| 97 | CCND1 |  |  | 8346 | -0.368 | -0.2052 | Yes |
| 98 | BAX |  |  | 8426 | -0.398 | -0.1963 | Yes |
| 99 | TSPO |  |  | 8443 | -0.404 | -0.1800 | Yes |
| 100 | FEZ1 |  |  | 8457 | -0.412 | -0.1631 | Yes |
| 101 | LMNA |  |  | 8478 | -0.423 | -0.1464 | Yes |
| 102 | PEA15 |  |  | 8509 | -0.439 | -0.1301 | Yes |
| 103 | LGALS3 |  |  | 8550 | -0.460 | -0.1141 | Yes |
| 104 | VDAC2 |  |  | 8564 | -0.466 | -0.0947 | Yes |
| 105 | HSPB1 |  |  | 8586 | -0.478 | -0.0757 | Yes |
| 106 | ANXA1 |  |  | 8595 | -0.484 | -0.0549 | Yes |
| 107 | CDKN1A |  |  | 8679 | -0.545 | -0.0399 | Yes |
| 108 | SOD1 |  |  | 8848 | -0.709 | -0.0271 | Yes |
| 109 | GPX4 |  |  | 8966 | -0.946 | 0.0020 | Yes |
Table: GSEA details [plain text format]

  

Fig 2: HALLMARK\_APOPTOSIS: Random ES distribution      
 Gene set null distribution of ES for **HALLMARK\_APOPTOSIS**
